# Effect of 8-week oral supplementation with 3-µg cyano-B12 or hydroxo-B12 in a vitamin B12-deficient population

**DOI:** 10.1007/s00394-017-1590-0

**Published:** 2017-12-05

**Authors:** Eva Greibe, Namita Mahalle, Vijayshri Bhide, Sergey Fedosov, Christian W. Heegaard, Sadanand Naik, Ebba Nexo

**Affiliations:** 1 Department of Clinical Biochemistry and Institute of Clinical Medicine, Aarhus University Hospital, Aarhus, Denmark; 2 Department of Pathology, Deenanath Mangeshkar Hospital and Research Center, Pune, India; 3 Department of Molecular Biology and Genetics, Aarhus University, Aarhus, Denmark

**Keywords:** Vitamin B12, Cobalamin, Cyano-B12, Hydroxo-B12, B12 supplementation, B12 deficiency

## Abstract

**Purpose:**

We compare the effect of 8-week oral supplementation with cyano-B12 (currently used in vitamin pills) and hydroxo-B12 (predominant form in the diet) in a population with nutritional vitamin B12 deficiency.

**Methods:**

Fifty-one healthy Indian adults with baseline serum cobalamin < 200 pmol/L were supplied for 8 weeks with daily oral supplements of 3-µg cyano-B12 (*n* = 15), 3-µg hydroxo-B12 (*n* = 16), or a placebo (*n* = 20). Blood at baseline, and each following week, was examined for total cobalamin, holotranscobalamin, methylmalonic acid, and homocysteine.

**Results:**

The study groups did not differ at baseline and were characterized by [median (range)] serum cobalamin [128 (68–191) pmol/L], holotranscobalamin [16 (6–41) pmol/L], methylmalonic acid [0.8 (0.3–1.7) µmol/L], homocysteine [17.9 (8.5–100.9) µmol/L], and a combined indicator of B12 status 4cB12 of − 1.65 (− 0.64 to − 4.07). The group supplemented with cyano-B12 showed a higher increase in total serum cobalamin than the group treated with hydroxo-B12, while other biomarkers changed comparably in the two groups. After 8 weeks of treatment, the biomarker values of the supplemented groups (pooled) differed significantly from the placebo group. Yet, the vitamin B12 status was still poor [cobalamin: 168 (87–302) pmol/L; holotranscobalamin: 19 (8–45) pmol/L; methylmalonic acid: 0.7 (0.2–1.7) µmol/L; homocysteine: 17.2 (2.6–96.8) µmol/L; 4cB12 = − 1.34 (− 0.33 to − 3.3)].

**Conclusion:**

8-week supplementation with 3-µg cyano-B12 elevated serum cobalamin more than 3 µg hydroxo-B12, but all other biomarkers changed similarly in both groups. Supplementation with 3 µg vitamin B12 did not reverse the low status in individuals with nutritional vitamin B12 deficiency.

**Clinical Trial Registry of India:**

REF/2017/02/013343.

**Electronic supplementary material:**

The online version of this article (10.1007/s00394-017-1590-0) contains supplementary material, which is available to authorized users.

## Introduction

Vitamin B12 (B12) is involved in a number of key metabolic processes including cell division and nervous system function. Deficiency may result in development of anemia and/or neurological symptoms. The vitamin is present in foods of animal origin as the coenzymes 5′-deoxyadenosyl-B12 and methyl-B12 [1] that both are converted into hydroxo-B12 (HO-B12) upon light exposure [2]. Thus, HO-B12 is a ubiquitous and sometimes predominant form of B12 in food [1]. The daily supply of B12 in a Western omnivorous diet is around 6 µg [3, 4], well above the recommended daily allowance (RDA) of 2.4 µg [5].

Populations of some countries—like India [6]—consume a diet containing B12 levels far below the RDA, and are, therefore, at risk of developing low B12 status and possibly overt B12 deficiency. Thus, during the last decade, there has been a growing awareness of the need for food fortification or supplementation of the diet with vitamin pills in such populations [7, 8]. Biochemically, B12 deficiency causes a decline in serum cobalamin (Cbl) and holotranscobalamin (holoTC); also called “active” B12, because this protein-B12 complex ensures transport of the vitamin into the cells. An increase in the metabolic markers methylmalonic acid (MMA) and/or homocysteine (Hcy) also indicates B12 deficiency. To combat this, the synthetic cyano-B12 (CN-B12) is used for supplementation purposes, because it is the least expensive vitamin form and resists aggressive chemicals better than HO-B12. Several studies have documented that both forms are retained alike after oral uptake [9–11], but the tissue distributions of the two absorbed vitamins differ. For example, in rats, considerably more B12 is internalized in the liver after ingestion of HO-B12, when compared to an equal dose of CN-B12 [12, 13]. Conversely, the circulating level of B12 is much higher upon administration of CN-B12 than after intake of similar dose of HO-B12 [11–14].

The current knowledge leaves us with two important questions: can a daily oral dose of B12 just above the RDA repair the B12 status of individuals not receiving sufficient quantities of dietary B12? Do treatments with either CN-B12 or HO-B12 lead to different or comparable results? Here, we investigate the effect of 8-week daily supplementation with 3 µg of CN-B12 or HO-B12 on markers of the B12 status in a B12-deficient Indian population.

## Subjects and methods

### Participants and study design

This longitudinal cohort intervention study was designed to investigate the B12 status in nutritionally induced B12-deficient Indian individuals, supplemented for 8 weeks with one daily dose of 3-µg CN-B12, 3-µg HO-B12, or no B12 (placebo). Non-fasting blood samples were drawn at baseline and each week throughout the study. Collected samples were measured for Cbl and related variables (see Biochemical measurements). The study was blinded, so that participants and health care staff did not know the content of the administered capsules.

Participants aged ≥ 18 years (*n* = 60) (27 males and 33 females) were recruited from the Pune area in India, and the study was carried out at Deenanath Mangeshkar Hospital and Research Center, Pune, India, in the spring of 2016. Most of the participants were healthy staff at the hospital. Inclusion criterion was a plasma Cbl below 150 pmol/L measured 2 weeks prior to enrollment. Exclusion criteria were use of vitamin pills containing > 1-µg B12 within the last 2 weeks, drugs known to influence B12 absorption, and any known chronic systemic disease. The number of participants included in the study was based on power calculations, using a multiple linear regression showing a statistical power of 90% (*α* = 0.05). The number of participants was adjusted for expected dropout due to the nature of the longitudinal design. The 60 participants were randomly divided into three groups [CN-group (*n* = 20), HO-group (*n* = 20), and placebo group (*n* = 20)] with an equal distribution of sex and age. All participants were asked to eat their usual diet throughout the study. Most of the participants were lacto-vegetarians (65%) and the rest were partly non-vegetarians (consuming a portion of non-vegetarian food approximately twice a week). The participants received no money for participation in the study; however, the placebo group received 8 weeks of B12 supplements (Neurobion with 15-µg CN-B12) for compensation after the study was completed.

### Preparation of CN-B12 and HO-B12 capsules for oral administration

Here and throughout the manuscript, the term HO-B12 covers both hydroxo-B12 and aquo-B12. The two forms are interchangeable, and their presence depends on the pH of the solution. CN-B12 and HO-B12 were given as aqueous solutions absorbed in 30-mg sugar and packed in two-piece hard-shell gelatin capsules (Natur-Drogeriet, Horning, Denmark) as described in Greibe et al. [14]. In brief, CN-B12 (Betolvex, A ctavis, Gentofte, Denmark) and HO-B12 (Vibeden, Sandox, Copenhagen, Denmark) were dissolved in sterile demineralized water and centrifuged at 12,000×*g* for 5 min at room temperature. The supernatants were retained, and the B12 concentrations were adjusted to 0.25 mg/mL in sterile demineralized water. To ensure that the concentrations were accurate, six aliquots of each B12 stock were diluted 1:5 in sterile demineralized water and converted to di-CN-B12 by incubating them with 0.5-mL 0.2-M KCN for 1 h in the dark. The absorption of di-CN-B12 at 368 nm was determined using a Shimadzu UV-1800 Spectrophotometer (Holm & Halby, Broendby, Denmark), and used to calculate the B12 concentration using a molar absorption coefficient of di-CN-B12 of 30,400 L/mol/cm [2]. The mean of six aliquots was used to calculate the exact stock volume needed to make capsules containing 3-µg CN-B12 and 3-µg HO-B12. The coefficient of variation (CV%) was 1% for the six aliquots of both CN-B12 and HO-B12. For further testing, the sugar-B12 capsules were redissolved in water and analyzed for B12 content using the Advia Centaur CP Immunoassay System (Siemens). The placebo capsules were made by packing 30-mg sugar in gelatin capsules without the addition of B12. All capsules, with or without B12, were stored in light-protected containers at 4 °C together with a desiccant. The maximal storage time of 6 weeks did not cause any changes in the B12 content, as previously demonstrated [14].

### Biochemical measurements

Blood samples were centrifuged (10 min at 2300×*g*) within 2 h of being drawn, and serum was stored at − 20 °C for later analysis. Serum aliquots were shipped to Denmark on ice for analysis of Cbl, holoTC, total transcobalamin (totalTC), total haptocorrin (totalHC), and MMA. The samples were frozen upon arrival. Hcy and hematological parameters (see below) were analyzed on EDTA plasma in India.

For each B12-related variable, all samples from each participant were measured in one run. Serum Cbl was measured on the Advia Centaur CP Immunoassay System (Siemens). Serum holoTC was determined using in-house sandwich ELISA, after removal of unsaturated transcobalamin (apoTC) with B12-coated magnetic beads [15]. The total imprecision was 8% [15] and the intra-assay imprecision was 4% [16]. The mean holoTC values for the low, intermediate, and high controls were 40, 70, and 114 pmol/L, respectively [16]. The samples were also examined for totalTC using the above-mentioned TC ELISA. TotalHC was measured by in-house HC ELISA with a total imprecision of 5% and the intra-assay imprecision of 2% [17]. The amount of B12 bound to HC (HC-B12) was calculated as total serum Cbl minus holoTC.

MMA was quantified by Liquid Chromatography–Tandem Mass spectrometry on the AB SCIEX Triple Quad 5500 System (AB SCIEX). Hcy was measured on the Architect Immunoassay Analyser (Abbott). Hemoglobin (Hb) and red blood cell mean cell volume (MCV) were determined on the XN 3000 Hematology Analyzer (Sysmex), and plasma creatinine was measured on the RX Imola (Randox Laboratories), employing routine ISO certified assays. The combined indicator of B12 status, 4cB12, was calculated from the measurements of serum Cbl, holoTC, MMA, and Hcy using the formula presented by Fedosov et al. [18].

### Statistics and data fitting

**Fig. 1:**
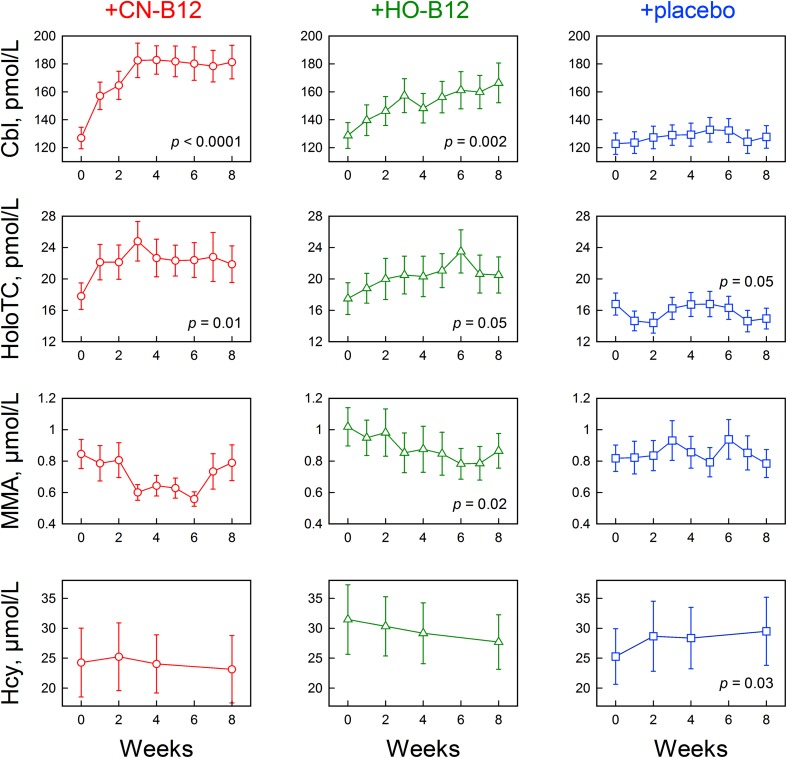
Changes in markers of B12 status in response to low-dose B12 supplementation. Indian adults with low B12 status received daily oral supplementation with 3-µg CN-B12 (*n* = 15); 3-µg HO-B12 (*n* = 16); or placebo (*n* = 20) for 8 weeks. Absolute serum concentrations of cobalamin (Cbl), holotranscobalamin (holoTC), methylmalonic acid (MMA), and homocysteine (Hcy) are shown (mean ± SE). Statistical differences between baseline and week 8 are indicated (paired *t* test). *p* values ≤ 0.05 were accepted as statistically significant. The figure is made in KyPlot version 5.0

The D’Agostino-Pearson omnibus test was used to determine if data followed the Gaussian distribution. The unpaired *t* test (normally distributed data) or the Mann–Whitney *U* test (not normally distributed data) was used to test for differences between the groups at individual time points, and the paired *t* test (normally distributed data) or the Wilcoxon’s signed-rank test (not normally distributed data) was used to test for differences between baseline and individual time points within the groups (Table 1; Fig. 1).

**Table 1:** Biomarker values recorded at baseline, and after 8 weeks of supplementation with a daily dose of 3-µg CN-B12, 3-µg HO-B12, or no supplementation (placebo)

	Ref. Int.	CN-B12 *n* = 15 (10 females)		HO-B12 *n* = 16 (10 females)		Placebo *n* = 20 (13 females)	
Age (years)	–	31 (22–42)		31 (22–50)		29 (21–48)	
		Baseline	8 weeks	Baseline	8 weeks	Baseline	8 weeks
4cB12	− 0.5 to 1.5	− 1.6 (− 1.1 to − 3.0)	− 1.3 (− 0.6 to − 2.7)	− 1.7 (− 0.8 to − 2.9)	− 1.7 (− 0.3 to − 2.6)	− 1.7 (− 0.6 to − 3)	− 2.0 (− 0.7 to − 2.9)
Cbl (pmol/L)	200–600	133 (68–171)	172* (99–262)	127 (68–191)	155* (87–302)	124 (68–190)	116 (85–191)
HoloTC (pmol/L)	40–150	18 (7–30)	20* (8–45)	16 (8–41)	19* (9–41)	16 (6–27)	14* (7–25)
TotalTC (pmol/L)	600–1500	805 (605–1190)	780 (630–1260)	890 (700–1235)	900 (670–1080)	878 (610–1370)	855 (625–1275)
TotalHC (pmol/L)	240–680	720 (540–2370)	570* (420–900)	750 (495–975)	600* (450–855)	788 (390–1350)	510* (360–855)
HC-B12 (pmol/L)	60–400	115 (61–148)	153* (87–240)	108 (54–175)	136* (74–278)	107 (56–173)	106* (74–174)
MMA (µmol/L)	0.1–0.3	0.8 (0.5–1.6)	0.7 (0.2–1.7)	1.2 (0.3–1.7)	0.9* (0.2–1.6)	0.8 (0.3–1.6)	0.7 (0.3–1.6)
Hcy (m) (µmol/L)	6.3–15.7	14.9 (13.3–35.8)	16.7 (12.9–39)	50.3 (12.3–86.8)	34.7 (18.3–74.8)	29.6 (9.6–94.5)	41.4 (13.4–116)
Hcy (f) (µmol/L)	4.9–14.9	17.9 (10–100)	16.0* (2.6–96.8)	14.7 (8.5–39.3)	13.6 (9.2–38.3)	14.2 (9.1–39.7)	17.0 (7.8-33-3)
Crea (m) (µmol/L)	60–105	96 (72–107)	–	89 (68–100)	–	80 (74–101)	–
Crea (f) (µmol/L)	45–90	76 (70–98)	–	74 (57–91)	–	73 (68–80)	–
Hb (m) (mmol/L)	8.1–10.3	9.1 (8.2–9.8)	–	9.5 (8.3–10.2)	–	9.2 (7.6–9.6)	–
Hb (f) (mmol/L)	7.1–9.3	7.8 (6.1–9.6)	–	7.6 (6.8–7.1)	–	8.1 (5.8–8.9)	–
MCV (fL)	82–98	86 (77–86)	–	86 (67–115)	–	86 (67–115)	–

Median with (range) is indicated. Reference intervals are from [15, 17–22]. No statistical difference in any parameter was found between the three groups at baseline (unpaired *t* test). The findings of low serum Cbl and holoTC concentrations combined with high MMA and Hcy concentrations confirm that the population has a low baseline B12 status, which is also supported by a combined indicator of B12 status 4cB12. Statistical differences (*p* ≤ 0.05) between baseline and after 8 weeks of treatment (paired *t* test) are denoted with asterisks. Serum Cbl, holoTC, and HC-B12 increased in response to the treatment with CN-B12 and HO-B12, and a small decline was observed for MMA in the HO-group, *p* = 0.02). Crea, Hb, and MCV were only measured at baseline. Results of Hcy, crea, and Hb were separated by males (m) and females (f)

*4cB12* combined indicator of B12 status; *Cbl* cobalamin; *holoTC* holotranscobalamin; *totalTC* total transcobalamin; *totalHC* total haptocorrin; *HC-B12* B12 bound to haptocorrin, calculated as total serum Cbl minus holoTC; *MMA* methylmalonic acid; *Hcy* homocysteine; *crea* creatinine; *Hb* hemoglobin; *MCV* red blood cell mean volume

**Fig. 2:**
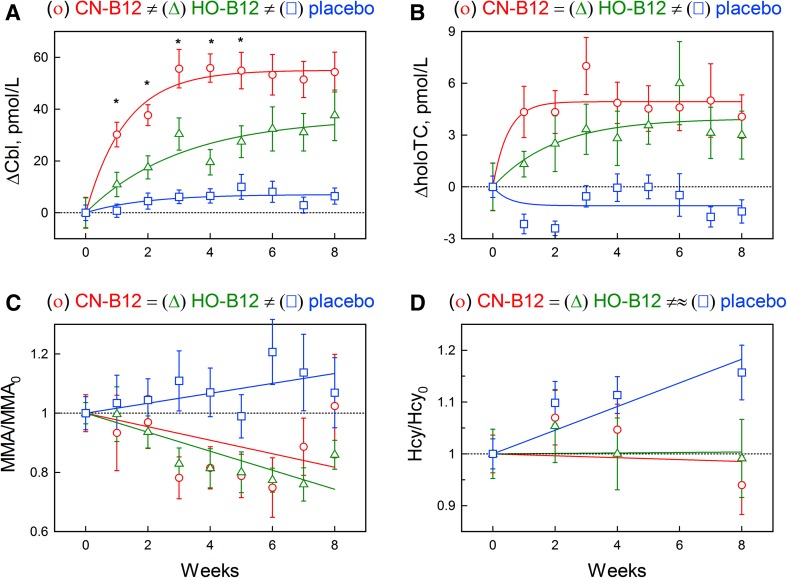
Normalized effect of CN-B12 and HO-B12 supplementation (adjusted for baseline). Change in **a** serum cobalamin (∆Cbl) and **b** holotranscobalamin (∆holoTC); relative change in **c** methylmalonic acid (MMA/MMA_0_) and **d** homocysteine (Hcy/Hcy_0_). Statistical difference between the CN-group and the HO-group at each individual time point is indicated with an asterisk (unpaired *t* test). The experimental points are presented as means ± SE for treatments with CN-B12 (circle, red), HO-B12 (triangle, green), and placebo (square, blue). The solid lines show fitting models obtained after approximation of all points available. The dotted line indicates the baseline level. The overall comparison of the models (unpaired *t* test) is schematically shown above each subfigure; see main text and Supplementary Data S1 for the details. The figure is made in KyPlot version 5.0

Changes in serum Cbl (ΔCbl = Cbl−Cbl_0_) and holoTC (ΔholoTC = holoTC−holoTC_0_) from the respective baselines (*X*_0_) were calculated for each patient (Fig. 2). The data were plotted over time as three data sets (CN-group, HO-group, and placebo group), and the points for each group were fitted using an exponential function. The underlying mathematics is presented in Supplementary data S1. The fitted models for each group were compared with *t* tests.

Changes in MMA and Hcy over time were presented as ratios between the concentration at a given time point and the concentration at the baseline (e.g., MMA/MMA_0_). Difference from the baseline (ΔMMA and ΔHcy) could not be used, because this value is proportional to the baseline concentration (MMA_0_ and Hcy_0_) (see the theoretical substantiation of this statement in [23]). At a limited concentration interval, the dependence on baseline can be compensated by division (X/X_0_). The ratios were plotted as three data sets (for CN-group, HO-group, and placebo group) and fitted by a linear function. The underlying mathematics is presented in Supplementary data S1. The fitted models for each group were compared with *t* tests.

Data analysis was performed using GraphPad Prism 5 and KyPlot 5.0 software.

## Results

### Characteristics of the study population

Indian participants (*n* = 60) with pre-study serum Cbl below 150 pmol/L (measured 2 weeks prior to enrollment) were divided into three groups: the CN-group (CN-B12 supplementation), the HO-group (HO-B12 supplementation), and the placebo group (no B12 supplementation). The median (range) age of the participants (27 males and 33 females) was 30 [21–50] years. Six participants dropped out of the study right after inclusion; four from the CN-group and two from the HO-group. One participant from the placebo group dropped out after 6 weeks of treatment, but the collected data from the first 6 weeks were included in the study. One participant in the HO-group presented extreme values at baseline (serum Cbl: 72 pmol/L; holoTC: 4 pmol/L; MMA: 5.4 µmol/L; Hcy: 116 µmol/L). He was excluded from the final data set, but his biomarker values are presented in Supplementary Data S2. Finally, we excluded two participants (one from the CN-group and one from the HO-group) with baseline serum Cbl levels well above the lower limit (200 pmol/L) of the reference interval [19] (240 and 340 pmol/L, respectively).

The baseline characteristics of the three groups (Table 1) did not differ, and all showed metabolic signs of B12 deficiency. All baseline values of the markers (serum Cbl, holoTC, MMA, Hcy) deviated considerably from the reference intervals in Western populations. The combined indicator of B12 status (4cB12 = [median (range)] − 1.65 (− 0.64 to − 4.07)] corresponded to the overall score of deficiency − 2 ± 0.5 (classified as possible B12 deficiency) [18]. Two participants showed extremely high levels of Hcy ≥ 100 µM, which may be the result of a joint B12 and folate deficiency (folate not measured).

The baseline correlations showed that (1) serum Cbl was strongly inversely correlated to both MMA (*r* = − 0.42, *p* = 0.002) and Hcy (*r* =− 0.55, *p* < 0.0001); (2) MMA and Hcy were related to each other (*r* = 0.31, *p* = 0.03); (3) MCV was related to Hcy (*r* = 0.40, *p* = 0.004), but not to serum Cbl or MMA concentrations.

The baseline concentration of totalHC (Table 1) is above reference intervals (determined for a Scandinavian population [17]); but at the same level of a comparable Indian population with low B12 status [14].

### Effect of intervention

The participants received one daily dose of 3-µg CN-B12 (CN-group), 3-µg HO-B12 (HO-group), or no supplementation (placebo group) for 8 weeks. Figure 1 shows the absolute measurements of the biomarkers of B12 status at each time point, and Fig. 2 shows the model approximations of the normalized changes expressed as differences from the baseline for serum Cbl and holoTC (Fig. 2a, b) or ratios to the baseline for MMA and Hcy (Fig. 2c, d), as explained in the method section and in the Supplementary Data S1. The participants with high baseline MMA and Hcy showed a larger decrease in metabolite concentration in response to B12 supplementation when compared with the participants with lower baseline values (cut-off MMA = 0.7 µmol/L, *p* = 0.003; cut-off Hcy = 20 µmol/L, *p* < 0.0001). Though not included in this calculation, this was even more obvious for the participant with highly elevated baseline levels of both MMA and Hcy (Supplementary Data S2). The observed proportionality of response to the baseline was, at least partially, compensated for using ratios (MMA/MMA_0_ and Hcy/Hcy_0_) instead of differences.

At the first stage of analysis, each group was examined for response during the time of treatment. This comparison included a paired *t* test of baseline (week 0) vs. weeks 1–8, as well as examination of the fitting models “test” vs. “zero” (i.e., probability that all floating parameters in Eq. 1 or Eq. 2 are equal to zero, see Supplementary Data S1).

The placebo group showed no change in the absolute values of serum Cbl or MMA during the study period, but revealed a small decline in holoTC (from 16 to 14 pmol/L; *p* = 0.05) and a small increase in Hcy (from 25.3 to 29.5 µmol/L; *p* = 0.03), when testing week 8 vs. week 0 (Fig. 1). The model approximations of the normalized placebo data (Fig. 2, square-blue) indicated a small but detectable upward shift in ΔCbl (*p* = 0.01). An upward drift of Hcy/Hcy_0_ was also detected (*p* = 6 × 10^− 4^). Possible decrease of ΔholoTC (*p* = 0.10) and increase of MMA/MMA_0_ (*p* = 0.16) were less convincing. It can be conjectured that the metabolic state of the placebo group has a tendency to deteriorate in terms of the overall B12 status.

The CN-group showed an overall increase in serum Cbl (from 133 to 172 pmol/L; *p* < 0.0001) and holoTC (from 18 to 20 pmol/L; *p* < 0.0001) in response to 8 weeks of supplementation (Fig. 1; Table 1). The approximating fits of ΔCbl and ΔholoTC (Fig. 2a, b, circle-red) also differed considerably from the respective “zero” models (probabilities of the latter were assessed as *p* = 10^− 17^ and 0.0004, respectively). The increasing curves apparently leveled off after 5 weeks for ΔCbl and 2 weeks for ΔholoTC (all numerical fitting coefficients are presented in Supplementary Data S1). A small decline in the absolute concentration of MMA was observed after 3 weeks of supplementation (*p* = 0.01), but this change was no longer significant after 8 weeks (Fig. 1). Yet, no significant difference between weeks 3 and 8 was found either. No change in the absolute concentrations of Hcy was found during the study period (Fig. 1). Figure 2c (MMA/MMA_0_) and Fig. 2d (Hcy/Hcy_0_), circle-red, show the fitted curves of normalized data. Responses to CN-B12 supplementation (i.e., linear slopes *A*_2_ in Eq. 2, Supplementary Data S1) were either uncertain (MMA/MMA_0_, *p* = 0.08) or clearly absent (Hcy/Hcy_0_, *p* = 0.80).

The HO-group showed an increase in both serum Cbl (from 126 to 154 pmol/L; *p* = 0.001) and holoTC (15 to 18 pmol/L; *p* = 0.001) in response to the supplementation. The same tendency was exhibited in the normalized models for ΔCbl (*p* = 10^− 6^) and ΔholoTC (*p* = 0.003). The respective functions apparently reached plateaus at the end of treatment, and the process developed somewhat faster for ΔholoTC (Fig. 2a, b, Z-green). The absolute concentrations of MMA showed a decline after 3 weeks of treatment (*p* = 0.04), and the change remained significant for the rest of the study period (*p* = 0.02), whereas no changes in Hcy were observed (Fig. 1). The normalized chart of MMA/MMA_0_ showed a tendency to decline (*p* = 3 × 10^− 5^), whereas Hcy/Hcy_0_ apparently remained on the same level (*p* = 0.10), Fig. 2c, d, Z-green.

At the second stage of analysis, we compared the responses between the three groups. This was done by a pairwise examination of the fitting models (e.g., “CN-group” vs. “HO-group”, “CN-group” vs. “placebo group”, etc), where the identity of all complimentary fitting parameters between groups “A” vs. “B” was tested (see Supplementary data S1).

Both treatment groups considerably differed from the placebo group in their ΔCbl records (*p* = 10^− 10^ for CN-group = placebo group and *p* = 0.0009 for HO-group = placebo group) and ΔholoTC records (*p* = 0.0003 for CN-group = placebo group and *p* = 0.003 for HO-group = placebo group), Fig. 2a, b. Treatment groups also differed from each other in ΔCbl. For example, the CN-group showed both a steeper increase in ΔCbl and a higher plateau level compared with the HO-group (*p* = 0.01 for CN-group = HO-group), Fig. 2a. The two treatment models apparently did not differ in ΔholoTC (*p* = 0.34 for CN-group = HO-group). The fraction of total Cbl attached to HC (HC-Cbl) increased similar to the total Cbl in CN- and HO-groups (data not shown).

The normalized records of MMA/MMA_0_ demonstrated very similar linear slopes over time for both vitamin forms (*p* = 0.54 for CN-group = HO-group), which differed from the placebo group (*p* = 0.03 for CN-group = placebo group and *p* = 0.0006 for HO-group = placebo group). It should be noted in this regard that the combination of an “insignificant” upward drift in the placebo group with an “insignificant” downward drift of the CN-group provided a “significant” difference between the two records of MMA/MMA_0_ (Fig. 2c, square-blue vs. circle-red). Disregarding the apparently unchanging level of Hcy/Hcy_0_ in both treatment groups, both variants of the vitamin supplementation apparently differed from the upward-tending placebo group (*p* = 0.02 for CN-group = placebo group and *p* = 0.06 for HO-group = placebo group).

No major differences were observed between males and females (data not shown).

## Discussion

Here, we investigate and compare the effect of supplementation with one daily oral dose (3-µg) of CN-B12 (used in vitamin pills) or HO-B12 (found in food items) for 8 weeks on the B12 status in a nutritionally B12-deficient Indian population. We report two major findings: (1) supplementing with CN-B12 increases serum Cbl more than HO-B12, but the metabolic parameters of B12 status respond similar to both treatments; (2) supplementing for 8 weeks with 3-µg B12 per day causes only minor improvements in the metabolites, with slightly decreasing MMA and stabilizing Hcy concentrations.

Our study has some limitations. The number of participants was relatively small and the duration of the trial was only 8 weeks. For this reason, we cannot conclude on the effect of long-term treatment with low doses of CN-B12 and HO-B12. We did not test if our participants could absorb B12 prior to the study. However, based on the increase in holoTC and/or plasma B12 in response to treatment with HO-B12 and CN-B12, the participants were judged able to absorb B12. Despite these limitations, the study provides a proof-of-concept and stresses that larger long-term intervention studies are needed to evaluate if treatment with low doses of B12 can rescue a nutritionally induced low B12 status.

Currently, CN-B12 is the preferable form for oral use in vitamin pills, fortification, and pharmacological preparations. The rational is that this form of the vitamin is both chemically stable and cheap. In agreement with the previous studies [11–14], we find that CN-B12 induces a higher increase in the circulating total Cbl in comparison to HO-B12. Interestingly, administration of HO-B12 is as efficient—if not slightly better—in improving other markers of an impaired B12 status, notably MMA. This result is in accord with our previous studies in rats [12, 13]. Thus, based on our present data, we conclude that the increase in serum Cbl upon administration of CN-B12 does not mirror a superior effect of this vitamin form, but rather shows that CN-B12 has a tendency to fall behind HO-B12 in the rates of tissue uptake. Further studies are warranted to prove whether HO-B12 is even superior to CN-B12 in its biological effect.

In agreement with the previous works [6, 7, 24], we report that supplementation with 3 µg of either CN-B12 or HO-B12 for 8 weeks caused a minor improvement in biomarkers of B12 status. We observed a gradually reached steady state in serum Cbl (after 5–8 weeks of treatment) and holoTC (after 2–6 weeks) and only marginal changes in MMA and Hcy. However, at the end of the study, concentrations of all markers were still far below (serum Cbl and holoTC) and far above (MMA and Hcy) the reference intervals for Western populations. Two previous intervention studies were conducted in comparable populations from the same geographical region in India and showed similar results. Naik et al. [6] found that low doses of B12 (2.5–3-µg/day) (supplied for 2 weeks in buffalo milk increased plasma concentrations of Cbl and reduced concentrations of Hcy after 2 weeks of treatment, without reaching reference intervals. Yajnik et al. [7] found the same result when orally supplementing methyl-B12 as high as 500 µg/day for a period of 6 weeks. In the latter study, a plateau of 13-µmol/L Hcy was reached after 2 weeks of treatment. No further changes occurred within the next 4 weeks of treatment, and neither plasma Cbl nor Hcy reached the accepted reference levels within the 6 weeks of study. The authors rightfully speculated that the findings could be explained by saturation of the absorption system, giving a balance between the absorption rate and the tissue clearance disregarding the excessive dose of B12. Furthermore, Rajan et al. [24] found that a daily dose of 25 and 100-µg B12 could not normalize MMA and Hcy within 6 weeks in a population of B12-deficient elderly. On the other hand, Rajan et al. [24] and Morkbak et al. [25] both demonstrated that high pharmacological doses of B12 (above 1000 µg/day) normalized the levels of Hcy or MMA within 4–6 weeks in B12-deficient elderly and vegans, respectively. Altogether, our data and the previous intervention studies [6, 7, 24, 25] indicate that the daily dose of B12 supplementation needs to contain quantities far above the RDA of 2.4-µg/day, to restore the optimal biomarkers of B12 status in individuals with a low B12 status due to inadequate intake. An alternative would be a more frequent supplementation with low-dose B12. In this regard, our recent study on acute uptake of B12 [14] showed that supplementation with three doses of 3 µg/day of CN-B12/HO-B12 for 2 days caused at least a ninefold increase in holoTC [median (range)] [CN-B12: ΔholoTC = 23 (0–55) pmol/L, *n* = 20; HO-B12: ΔholoTC = 7 (− 10 to 29) pmol/L; *n* = 19], vs. 1 week of treatment with one dose of 3 µg/day CN-B12/HO-B12 (this study; baseline vs. week 1) (CN-B12: ΔholoTC = 2.5 [− 3 to 18] pmol/L, *n* = 20; HO-B12: ΔholoTC = 0.5 [− 3 to 7] pmol/L, *n* = 20).

An interesting finding of the present study is that the plasma concentrations of both MMA and Hcy apparently decrease after a lag period of 2–4 weeks, respectively, following administration of a low physiological dose of oral B12 on daily basis (Figs. 1, 2). Notably, in these lag periods, 60–70% of the participants showed a decrease in MMA and 30–35% in Hcy, whereas the rest belonged to “non-responders”. The number of participants responsive to treatment increased to 70–90% (MMA) and 50–70% (Hcy) during the following weeks. In other words, the treatment groups apparently separate into fast responders (showing a noticeable change soon after the beginning of treatment) and slow responders (showing a lag). This fact presents some difficulty in the description of metabolites vs. time by a specific mathematical function, e.g., exponent, line, sigmoid, etc. In the absence of more firm evidence, a linear approximation of the ratio records was used here (Fig. 2c, d). Another interesting fact concerns a single individual with high baseline levels of the metabolic markers. This participant showed the best initial response in Hcy, which was, however, not mirrored in MMA (Supplementary Data S2). The latter metabolite started to decrease only after a lag of 2 weeks. The biological rationale of such difference might imply slower accumulation of the cofactor in mitochondria (MMA reaction) in comparison to the cytoplasm (Hcy reaction). The previous studies have shown that high doses of injected B12 decreased MMA within a few days [26]. We speculate that the time of response might be dose-dependent, so that higher doses of B12 or/and frequent administration will shorten the lag period.

One study, apparently conflicting with the aforementioned studies and their conclusions should, however, be mentioned. Deshmukh et al. [27] observed a marked effect of 4-month treatment with 2-µg B12/day, which decreased Hcy in an Indian population to the values within the reference interval. Our population had lower baseline serum Cbl (128 pmol/L), but similar Hcy (18.1 µmol/L) in comparison to the Deshmukh population (Cbl; 166 pmol/L; Hcy: 18.6 µmol/L). We found almost no change in Hcy after 1.5 month of treatment, which could indicate that the response of metabolites to low doses of Cbl might be a sigmoid function with a pronounced lag period followed by a rather steep return to the normal level. Alternatively, the undetected “insignificant” changes (tested after a relatively short treatment period) can gradually lead to a final “significant” alteration of the metabolic balance. Further work is needed to address this issue.

In conclusion, we have demonstrated that 8 weeks of daily oral treatment with 3-µg CN-B12 or 3-µg HO-B12 causes an increase in total serum Cbl and holoTC, plus minor changes in the metabolic markers MMA and Hcy. Our findings show that the current RDA is insufficient for a short-term restoration of an impaired B12 status. Future studies are requested to establish the dose of B12 needed to restore an impaired B12 status. A benefit of HO-B12 vs. CN-B12 administration was suggested, but not definitively proven. Further studies should be directed to explore the dose and the form of B12 requested to restore and maintain an optimal B12 status in populations with low dietary intake of this vitamin.

## Electronic supplementary material

Below is the link to the electronic supplementary material.

Supplementary material 1 (PDF 236 KB)

Supplementary material 2 (PDF 181 KB)

## Abbreviations

ApoTC: Unsaturated transcobalamin
B12: Vitamin B12
Cbl: Cobalamin
CN-B12: Cyano-B12
Hb: Blood hemoglobin
HC: Haptocorrin
HC-B12: B12 bound to HC
HO-B12: Hydroxo/aquo-B12
HoloTC: Holotranscobalamin
MCV: Mean cell volume
MMA: Methylmalonic acid
Hcy: Homocysteine
RDA: Recommended daily allowance
TotalHC: Total haptocorrin
TotalTC: Total transcobalamin
