# Supplementary material for: Effect of 8-week oral supplementation with 3-µg cyano-B12 or hydroxo-B12 in a vitamin B12-deficient population
Source: Eur J Nutr. 2017 Dec 5;58(1):261–70. doi: 10.1007/s00394-017-1590-0 (PMC6424936; doi:10.1007/s00394-017-1590-0)
Supplement: Supplementary file 1 — Supplementary material 1 (PDF 236 KB) [file 394_2017_1590_MOESM1_ESM.pdf]

ELECTRONIC SUPPLEMENTARY MATERIAL

Effect of eight weeks' oral supplementation with 3-µg cyano-B12 or hydroxo-B12 in a vitamin B12-deficient population

European Journal of Nutrition (EJON)

Eva Greibe[1*], Namita Mahalle[2], Vijayshri Bhide[2], Sergey Fedosov[3], Christian W. Heegaard[3], Sadanand Naik[2*], Ebba Nexo[1]

[1]Department of Clinical Biochemistry and Institute of Clinical Medicine, Aarhus University Hospital, Aarhus, Denmark (EG, EN); [2]Department of Pathology, Deenanath Mangeshkar Hospital and Research Center, Pune, India (NM, VB, SN); [3]Department of Molecular Biology and Genetics, Aarhus University, Aarhus, Denmark (SF, CWH).

*Address correspondence and reprint requests to Eva Greibe, Department of Clinical Biochemistry, Aarhus University Hospital, Aarhus, Denmark. Phone: +45 26 39 10 09. Email: greibe@clin.au.dk

ONLINE RESOURCES 1

Data fitting and comparison of the treatment models.

Changes in total serum Cbl (ΔCbl = Cbl – Cbl_0) and holoTC (ΔholoTC = holoTC – $holoTC_0$) from the respective baselines (X_0) were calculated for each patient (Fig. 2). The data were plotted over time as three datasets (CN-group, HO-group, and placebo group), and the points for each group were fitted using an exponential function:

$$y = A_1 + A_2 \cdot (1 - e^{-A_3 \cdot t}) \qquad \text{Eq. 1}$$

where y is the dependent variable (either ΔCbl or ΔholoTC); A_1 is the baseline value; A_2 is the maximal amplitude of change; A_3 is the rate constant of change; t corresponds to the time of

treatment (independent variable). The fitting procedure included three parameters: fixed $A_1 = 0$ and floating A_2 and A_3. The fixed zero parameter A_1 was retained in Eq. 1 (and the covariance matrix) because its error adds to the errors of A_2 and A_3 making their statistical estimates more realistic. The probability (p_i) of zero value for each parameter A_i was assessed by t-test, and the overall probability of "zero" model ($A_1 = A_2 = A_3 = 0$) was given as $p_1 \cdot p_2 \cdot p_3 = p_2 \cdot p_3$ ($p_1 = 1$ for the assigned $A_1 = 0$). The parameters A_1, A_2, A_3 of different groups were aligned and compared pairwise (e.g. $A_{2,CN}$ ± SE for CN-group vs. $A_{2,HO}$ ± SE for HO-group) and possible equality of the two values was assessed using t-test. The overall identity of the two models (e.g. CN-group vs. HO-group) was given as $p_1 \cdot p_2 \cdot p_3$.

Changes in MMA and Hcy over time were presented as ratios between the concentration at a given time point and the concentration at the baseline (e.g. MMA/MMA_0). Difference from the baseline (ΔMMA and ΔHcy) could not be used because this value is proportional to the baseline concentration (MMA_0 and Hcy_0). At a limited concentration interval, the dependence on baseline can be compensated by division (X/X_0). The ratios were plotted as three datasets (for CN-group, HO-group and placebo group) and fitted by a linear function. The choice was taken after the initially attempted fitting Eq.1, which gave the curves of a nearly linear shape (not shown). The used function was recorded as follows:

$$y = A_0 + A_1 \cdot t \qquad \text{Eq. 2}$$

where y is the ratio (dependent variable); A_0 is the baseline value (assigned as 1); A_1 is the slope (floating parameter); t is the time. The approach to analysis of the fits was identical to the procedure for Eq. 1, expect for $A_1 = 1$ for a "zero" model.

Table S1. Parameters of the fitting models (approximating relative responses to treatments with CN-B12, HO-B12, and placebo, Fig. 2).

Marker response	CN-B12 Treatment	p_i of A_i = 0 or 1	HO-B12 treatment	p_i of A_i = 0 or 1	placebo treatment	p_i of A_i = 0 or 1
Cbl	Eq. 1		Eq. 1		Eq. 1	
A_1 ± SE, (p_1)	0.0 ± 6.0	(1)	0.0 ± 5.8	(1)	0.0 ± 3.0	(1)
A_2 ± SE, (p_2)	55.0 ± 6.4	($3\cdot10^{-14}$)	36.7 ± 8.4	($2\cdot10^{-5}$)	7.1 ± 3.3	(0.035)
A_3 ± SE, (p_3)	0.78 ± 0.22	($6\cdot10^{-4}$)	0.33 ± 0.21	(0.11)	0.53 ± 0.64	(0.41)
(p, overall)		**($2\cdot10^{-17}$)**		**($2\cdot10^{-6}$)**		**(0.014)**
holoTC	Eq. 1		Eq. 1		Eq. 1	
A_1 ± SE, (p_1)	0.0 ± 1.4	(1.0)	0.0 ± 1.4	(1.0)	0.0 ± 0.7	(1.0)
A_2 ± SE, (p_2)	4.9 ± 1.5	(0.0011)	4.0 ± 1.5	(0.011)	–1.2 ± 0.75	(0.14)
A_3 ± SE, (p_3)	2.1 ± 2.3	(0.38)	0.50 ± 2.3	(0.32)	2 ± 5	(0.69)
(p, overall)		**(0.0042)**		**(0.0036)**		(0.10)
MMA	Eq. 2		Eq. 2		Eq. 2	
A_1 ± SE, (p_1)	1.0 ± 0.06	(1.0)	1.0 ± 0.03	(1.0)	1.0 ± 0.06	(1.0)
A_2 ± SE, (p_2)	–0.022 ± 0.013	(0.082)	–0.032 ± 0.007	($3\cdot10^{-5}$)	0.017 ± 0.011	(0.16)
(p, overall)		(0.082)		**($3\cdot10^{-5}$)**		(0.16)
Hcy	Eq. 2		Eq. 2		Eq. 2	
A_1 ± SE, (p_1)	1.0 ± 0.04	(1.0)	1.0 ± 0.05	(1.0)	1.0 ± 0.03	(1)
A_2 ± SE, (p_2)	–0.002 ± 0.008	(0.82)	0.00 ± 0.01	(0.96)	0.023 ± 0.006	($6\cdot10^{-4}$)
(p, overall)		(0.82)		(0.96)		**($6\cdot10^{-4}$)**

Table S2. Probability of equal fitting models for CN-B12 vs. HO-B12 treatments and each treatment vs. placebo (Fig. 2).

Marker	*p* of CN-B12 = HO-B12	*p* of CN-B12 = placebo	*p* of HO-B12 = placebo
ΔCbl	**0.011**	$\mathbf{1.6 \cdot 10^{-10}}$	$\mathbf{8.7 \cdot 10^{-4}}$
ΔholoTC	0.34	$\mathbf{3.3 \cdot 10^{-4}}$	**0.0026**
MMA/MMA_0	0.54	**0.025**	$\mathbf{5.8 \cdot 10^{-4}}$
Hcy/Hcy_0	0.86	**0.016**	<u>0.067</u>
