# Supplementary material for: Effect of 8-week oral supplementation with 3-µg cyano-B12 or hydroxo-B12 in a vitamin B12-deficient population
Source: Eur J Nutr. 2017 Dec 5;58(1):261–70. doi: 10.1007/s00394-017-1590-0 (PMC6424936; doi:10.1007/s00394-017-1590-0)

ELECTRONIC SUPPLEMENTARY MATERIAL

Effect of eight weeks' oral supplementation with 3-µg cyano-B12 or hydroxo-B12 in a vitamin B12-deficient population

European Journal of Nutrition (EJON)

Eva Greibe[1*], Namita Mahalle[2], Vijayshri Bhide[2], Sergey Fedosov[3], Christian W. Heegaard[3], Sadanand Naik[2*], Ebba Nexo[1]

[1]Department of Clinical Biochemistry and Institute of Clinical Medicine, Aarhus University Hospital, Aarhus, Denmark (EG, EN); [2]Department of Pathology, Deenanath Mangeshkar Hospital and Research Center, Pune, India (NM, VB, SN); [3]Department of Molecular Biology and Genetics, Aarhus University, Aarhus, Denmark (SF, CWH).

*Address correspondence and reprint requests to Eva Greibe, Department of Clinical Biochemistry, Aarhus University Hospital, Aarhus, Denmark. Phone: +45 26 39 10 09. Email: greibe@clin.au.dk

ONLINE RESOURCES 2

Fig. S1 Biomarkers for a male participant (48 years) presenting extreme values at baseline. Serum cobalamin (Cbl), holotranscobalamin (holoTC), methylmalonic acid (MMA), and homocysteine (Hcy) are shown at baseline and during eight weeks of supplementation of 3-µg/day HO-B12. Hcy was only measure at baseline and at week 2, 4, and 8. Reference intervals (Ref. Int.) are indicated in the lower right corner in the same units as indicated on the y-axis. Note that the y-axis for Hcy does not start at zero. At baseline the biomarker values were: serum Cbl 72 pmol/L; holoTC: 4 pmol/L;

MMA: 5.4 µmol/L; and Hcy: 116 µmol/L. After eight weeks of treatment the values were: serum Cbl 108 pmol/L; holoTC: 7 pmol/L; MMA: 3 µmol/L; and Hcy: 92 µmol/L. The data from the participant was excluded from the final dataset presented in the main paper. However, the response pattern of the participant was comparable to the entire group though more extreme and in addition showing a decline in Hcy. Notable also for this participant, MMA declined only after three weeks of supplementation. The figure is made in KyPlot version 5.0

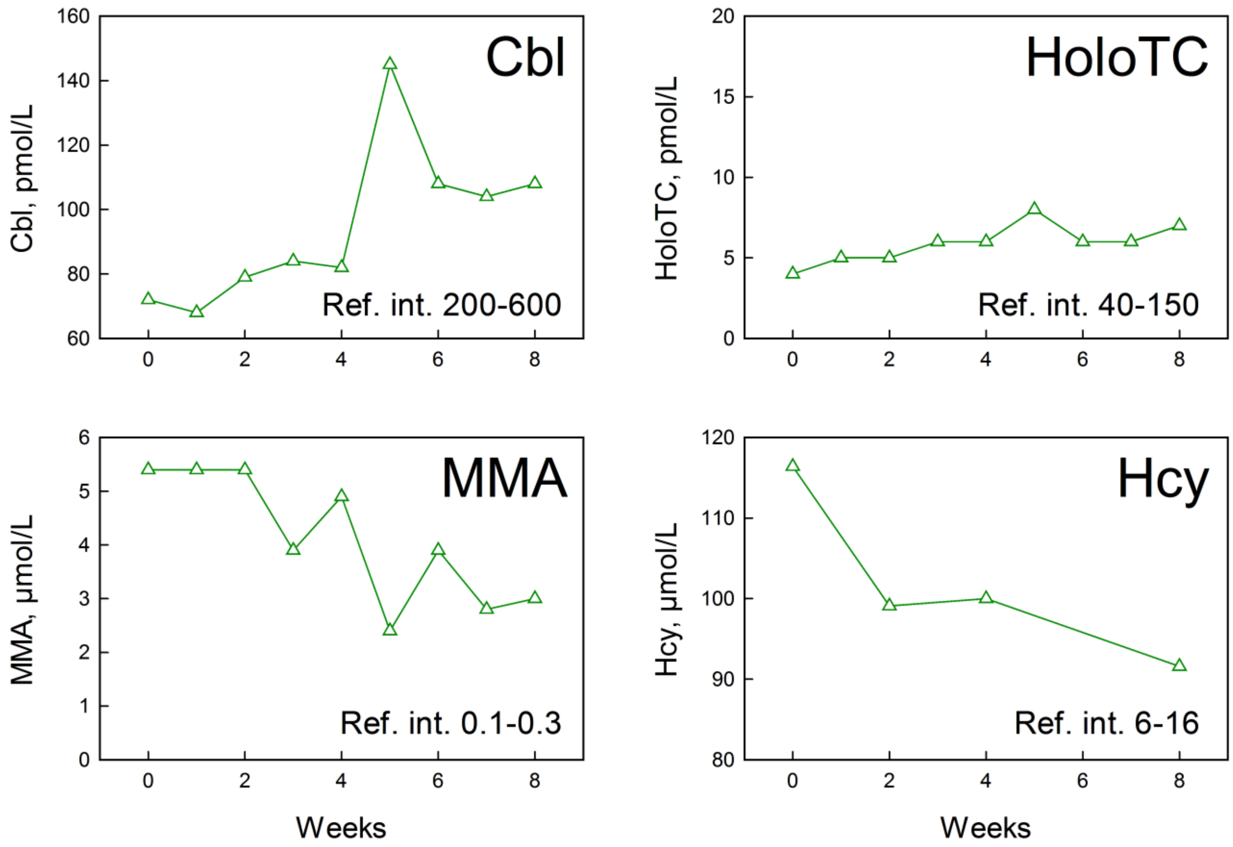

Supplement: Supplementary file 2 — Supplementary material 2 (PDF 181 KB) [file 394_2017_1590_MOESM2_ESM.pdf]
